# The influence of different curriculum designs on students’ dropout rate: a case study

**DOI:** 10.1080/10872981.2018.1432963

**Published:** 2018-02-02

**Authors:** John Vergel, Gustavo A. Quintero, Andrés Isaza-Restrepo, Martha Ortiz-Fonseca, Catalina Latorre-Santos, Juan Mauricio Pardo-Oviedo

**Affiliations:** ^a^ School of Medicine and Health Sciences, Medical and Health Sciences Education Research Group, Universidad del Rosario, Bogotá, Colombia; ^b^ School of Medicine and Health Sciences, Universidad del Rosario, Hospital Universitario Mayor, Bogotá, Colombia

**Keywords:** Dropout rate, curriculum development, medical education

## Abstract

The relationship between students’ withdrawal and educational variables has generated a considerable number of publications. As the explosion of information in sciences and integration theories led to creating different curriculum designs, it has been assumed that differences among designs explain academic success and, therefore, students’ retention. However, little attention has been given to examine explicitly how diverse designs influence dropout rates in practice, which questions if decisions to reform curricula are sufficiently informed. This article describes our curriculum reform, which exposes our former and current curriculum designs as having had dissimilar dropout percentages. Furthermore, we aimed to explore the influence of different curriculum designs on students’ dropout rates. The conclusion is that dropout variations may be explained not only because of the curriculum design itself, but also because of the power relationship changes between teachers and students that brought out the design change. Consequently, more research is needed to fully understand the political implications of different curriculum designs and their influence on dropout rates.

## Introduction

A central concern of any medical school is whether or not the curriculum in use is appropriate to ensure students’ academic success [1,2]. When students struggle academically, the probability of dropping out increases [3]. Therefore, understanding the influence of the educational variables, such as the type of curriculum design, on academic failure is of key importance to actively prevent dropout. However, although there is plenty of information about the association between dropping out and student attributes [4,5], little attention has been given to examining the influence of different curriculum designs – that, by the way, have become popular in medical education across the world during the last decades (e.g., PBL, integrated-system, spiral, transdisciplinary and interdisciplinary curriculum) – on students’ withdrawal [6,7].

The few studies that have examined the effects of different curriculum designs on the attrition rates show contradictory results, which create a gap in the literature around the value of different curriculum designs to prevent students’ withdrawal. For example, Iputo and Kwizera [8] compared the attrition patterns between traditional and problem-based learning curricula. They found the dropout rates to be significantly higher in the traditional curriculum. On the other hand, Strayhorn [9] found no significant differences. Because of this gap in knowledge and considering all the negative consequences of dropping out for society in general and health systems in particular [10], we believe it is necessary to understand how dissimilar curriculum designs influence the attrition rates in practice. Thus, curriculum designers (including teachers) may make informed decisions about which – and why – curriculum design is better to use to diminish students’ withdrawal when it comes to reforming the curriculum.

This paper reports a case study that aims to explore how different curriculum designs seem to affect the attrition percentages in different ways. We present the evidence and experience that we have had during the curriculum reform at Universidad del Rosario Medical School, Colombia. We want to continue the debate on the dropout rate and its relation to curriculum [11]. The implications for readers are that decreasing dropout rates are not only a result of the structural differences among diverse curriculum designs but also the influence that diverse designs have on changing power relationships between teachers and students. The following is the story of our curriculum change and its relation to students’ dropping out.

### The traditional curriculum

Walking through the main building of Universidad del Rosario is a trip into the distant past. The paintings of the historical figures and the colonial architecture break through the cosmopolitan environment of downtown Bogota. History and tradition comprise the signature of the university and a reason for students and faculty members to feel proud. When we refer to the traditional curriculum, we are not taking into consideration the historical values of the university but the conventional ways to design and implement a curriculum – which is disciplined-centered. Specifically, around 1802 a curriculum paradigm shift occurred. Medical curriculum tradition changed from the Galenic practices of the medieval period to the European medicine practices of the eighteenth century when José Celestino Mutis – a Spanish priest, botanist and physician – designed a new medical curriculum [12]. In spite of changes such as the adoption of the discipline-centered perspective and medical training at the patient’s bedside, the curriculum practice continued to have a hierarchical and vertical relationship between teachers and students, in which the teacher is placed above the student in relation to knowledge and skills.

With some interruptions caused by the independence of what today is Colombia from Spain (conflict period,1810–1818), the Mutis curriculum was implemented until 1865 when all medical teachers quit their jobs because of an economic crisis and Rosario Medical School was closed again for a hundred years [12]. The Medical School was reopened in 1965 after an agreement of collaboration between Universidad del Rosario and the Sociedad de Cirugía de Bogotá [13] (Bogota Surgeons’ Society). Guillermo Ferguson (pathologist) and Juan Didoménico (surgeon) redesigned the traditional curriculum combining the Flexnerian model (i.e., 2 years of basic sciences + 2 years of clinical sciences) with the European curriculum model (i.e., training at the patient’s bedside from day 1) yet continuing with the discipline-centered perspective [13]. Quevedo and Pérez [13] describe the structure of this curriculum as follow:

- The curriculum is organized around 12 academic periods (i.e., six months each academic period or 6 years) and is designed as a study plan constructed around topics, similar to the chapters of a medical textbook.
- During the 1st to 4th semesters, students study basic and biomedical sciences, while in the 5th and 6th semesters, students are dedicated to the study of clinical sciences.
- During the 7th to 10th semesters, students are immersed in clinical settings and rotate each 22 weeks among different clinical departments.
- The internship takes place during remaining semesters.
- The instructional methods are discipline-oriented and teacher-centered, including lectures, cadaveric dissections, laboratory work, and clinical autopsies under the guidance of a pathologist, clinical and pathological presentations, bedside training, clinical setting teaching and teacher-led seminars.
- Biomedical or clinical experts design the assessment tools. These assessments emphasize testing students’ capability of memorization.

### The integrated curriculum

Each week, a young lady delivers the institutional newspaper, *Nova et Vetera* onto the teachers’ desks. ‘Ever Old, Ever New’ is the slogan of the university and the principle that drives its educational changes. A major change of the medical curriculum entailed moving from the discipline-centered perspective to the experience-centered perspective and curriculum integration. Although the meanings of curriculum integration in this context are multiple, the term mainly refers to interwoven sciences, learning experiences, and personal-social interests.

More specifically, the last curriculum change began in 2004, when experts in medical education – under the guidance of the Colombian government – evaluated the medical education program designed by Fergurson and Didoménico and made some suggestions to improve it for accreditation purposes. Although the traditional curriculum received the accreditation of high quality after this evaluation, the experts recommended reforming the curriculum to promote the integration of sciences and to incorporate the international training trends into the medical education. The experts made this recommendation again in a subsequent program evaluation in 2010; leading to the creation of a curriculum reform office in 2013 that designed, developed and implemented the current, integrated curriculum.

In order to standardize the curriculum, the reform committee followed the six step procedure proposed by Kern [14]. Using this approach, the committee redefined what we understood as the health-illness concept, moving from a ‘state’ of health and disease towards a dynamic process determined by biological, psychological, social, cultural and historical factors. Based on this theoretical framework and the needs in our local context, the committee constructed a profile of the physician we want to produce, which was translated into general and specific learning outcomes. The learning outcomes were mapped and connected with different learning experiences and assessment tools across the courses. The final step to standardize the curriculum included socializing the curriculum mapping with the other teachers that did not directly participated in the reform. This step gave feedback that allowed the committee to improve the curriculum standards. Quintero [15,16] (surgeon) led the implementation of the new curriculum reform, describing it as innovative because of the following characteristics:

- The curriculum is organized around two phases of six academic periods each, and is designed as a competence-based curriculum that includes general and specific learning outcomes.
- Each academic period is composed of 16 weeks (except in the 4th year when students are immersed in clinical settings; here an academic period constitutes 20 weeks).
- The core curriculum is implemented during the first phase and includes modified problem-based learning experiences that are organized around organ systems. The learning experiences are intended to integrate four sciences: Basic/Biomedical, Clinical, Socio-humanism, and Population Health Sciences. Small groups of students discuss varied problems guided by a facilitator to enquire the information from the multiples sciences needed to solve the problems. Students also have early contact with patients in the hospital. All these learning experiences are referred to as Integrative Learning Activities by System (ILAS).
- Additional to the ILAS, the curriculum’s first phase includes disciplinary-centered courses such as Biosciences, Socio-humanism, Human Body Structure, Function and Therapeutics, Primary Care, Genetics, Translational Medicine, Clinical Simulation, and Epidemiology. Lectures and laboratory work are the main learning methodology in these courses.
- In the second phase, students are primarily immersed in clinical settings. However, during their 5th year, students can select from four choices for deepening knowledge and skills such as clinical specialties, medical research, primary health care and a double program.
- The internship takes place during the 6th year.
- Biomedical, clinical and social sciences experts design the assessment tools collaboratively under the guidance of an expert in education, which allows validation of the instruments. The assessments emphasize testing students’ capability of memorization, but also knowledge integration. The tools to assess students’ learning are varied, including multiple-choice questions, open-ended questions, Mini-ECOES, simulations and performance assessment. However, the amount of assessment periods/sessions were dramatically reduced as in the traditional curriculum students had approximately two assessments each week.
- Creating the mentorship and peer tutoring programs.

Planning the current curriculum took about five years. This is a process we have not yet finished as we are still reviewing, correcting and updating the curriculum standards according to the difficulties and strengths found during its implementation. Implementing the integrated curriculum also involved a significant investment of resources by the University, both human and material: for example, building classrooms for small groups of students, setting the curriculum via Moodle or training teachers in active pedagogy. Moreover, the number of weeks in each academic period was reduced for students to be able to have extracurricular activities such as sports or mindfulness. Reducing the academic periods was a strategy to enable medical students to cope better with stress and the demands of medical education. However, tensions emerged among teachers because of this strategy as lectures were reduced dramatically. Similarly, although the initial intention of the curriculum reform was to implement ILAS exclusively, several tensions also emerged as teachers and students valued lectures as part of the university’s traditions. For these reasons, and taking into consideration teachers’ and students’ learning needs and conceptions, it was decided to combine ILAS with disciplinary courses, creating a hybrid curriculum.

Unfortunately, the sentiment concerning the resistance of several teachers to change their traditional role as providers of information represented one barrier for integrating the curriculum. This was often seen as a result of losing autonomy in curriculum design and assessment. In order to reduce the resistance to the new changes, several strategies were used to promote teachers’ engagement in the new curriculum such as creating new roles (e.g., mentor teacher) and enhancing transparency and accountability of learning outcomes across the curriculum. To avoid tensions during the curriculum transition, the medical program was divided into two parts: one part involved students from the former curriculum (these students will finish their studies in 2019) and the other part involved students from the new curriculum that began in 2013. The two students’ cohorts have developed their studies using different curricular designs with no interaction in the learning experiences.

Aware that the new students could also develop feelings of resistance towards the new changes, other strategies were included during curriculum implementation to ensure that these students coped better as regards the new teaching style. The strategies included reforming the admissions criteria (i.e., testing reading and writing competencies) and training in oral and writing communication skills. It was assumed that these skills were going to help students better adapt to collaborative learning methodologies.

### Dropout rate under the traditional curriculum

Isaza et al. [17] investigated the dropout and graduation delay rates of two cohorts of the former curriculum during 2003 and 2008. The authors define dropping out as leaving the program for more than two academic periods, that is, one year of studies. More specifically, a student has dropped out of Rosario Medical School when he/she: (a) fails a particular course three times, (b) obtains a grade average below 3.0 out of 5.0, (c) fails three or more courses in one academic period, and (d) fails three or more courses of the basic sciences cycle. Additional reasons for leaving the school without finishing one’s studies are disciplinary suspension and not notifying the University they are not going to matriculate for the academic period in the preestablished time.

The longitudinal dropout analysis reported that 47% of the students in the first cohort dropped out the program, same as 36% of the students in the second cohort (See Table 1). Interestingly, most of the students in both samples (74%) left the program during the first two years of studies. The authors also describe the factors associated with dropping out and found that academic failure was the main reason for leaving the program without finishing one’s studies.

**Table 1.** Students’ dropout percentage under the traditional curriculum.

Cohort	Number of incoming students	Number and percentage of studentswho dropped out of the program	Number and percentageof students who delayed their studies	Graduates on time
2003–1	87	41 (47%)	27 (31%)	19 (22%)
2003–2	88	32 (36%)	39 (44%)	17 (19%)

### Dropout rate in the integrated curriculum

After implementing the first three years of the curriculum reform, we noticed the dropout percentage had declined dramatically. We decided to collect information on official dropout percentages during this period. In analyzing the results, we considered it worthwhile to publish them even though the first cohort of students had not completed their six years of study. We wanted to compare findings on dropout percentages using the integrated and the traditional curricula. Although we had incomplete data for the second phase of the integrated curriculum, we believe students’ withdrawal will not increase significantly during this phase because students usually do not leave their studies during the clinical immersion.

The Academic Secretary’s Office, which is the department that manages students’ academic information, publishes the anonymous dropout data each semester. Table 2 shows the temporal dropout data of the first six cohorts of students enrolled in the integrated curriculum during 2013 and 2016. Specifically, students’ dropout percentages ranged from 0.8 to 8.3%. In the fourth cohort, for example, only 4 of one 448 students left medical school after three academic periods without having finished their studies.

**Table 2.** Students’ dropout percentage under the integrated curriculum.

Academic period/variables	2013–2	2014–1	2014–2	2015–1	2015–2	2016–1	Total
Number of the cohort	I	II	III	IV	V	VI	–
Number of academic periods studied from 2013 to 2016	6	5	4	3	2	1	–
Number of admitted students in each cohort	143	143	133	148	131	117	815
Number of students that dropped out of the medical school	4	5	11	4	2	1	27
Temporal dropout percentage	2.8%	3.5%	8.3%	2.7%	1.5%	0.8%	3.3%

## Discussion

Dropping out usually has negative consequences on social, personal, financial and educational aspects of students’ lives, constituting a reason of great concern in medical schools [10,18]. Since dropping out represents an important educational problem, plenty of information on calculating the percentage of admitted students that drop out of medical school without completing their studies exists in the available literature [4,19–22]. One interesting finding from this information is that the average percentages of dropping out varies among developed countries [3], which is only 11.1% normally, and for developing countries such as those in South America [17], dropout rates may range from 43 to 62%. As a high attrition rate may entail a loss of social impact and potential contribution on enhancing health workplaces [10], which in turn anchor the development in poor countries, understanding medical school dropout rates and their associated factors are therefore of key importance in medical education research. The insights derived from this research may lead to future interventions for students at risk of dropping out, thus, increasing graduation rates.

Aware of the need to better understand medical school dropout rates, we examined how the average dropout percentages changed for two different curriculum designs in our medical school. The findings from this case study showed that there was an overwhelming decrease in dropout percentages after changing from one design to the other. While in the traditional curriculum sample an average of 41.5% of the admitted students dropped out of medical school before graduation [17], in the integrated curriculum sample the average was only 3.3%. Although students in the integrated curriculum have not yet finished their studies, a reality which limits a comparison of both curricula, we believe the dropout percentages during the remaining years of studies will continue stable. Nevertheless, considering only the first three years of studies between both samples, which are comparable to each other in study duration, the difference in the average of dropout percentages between both curriculum designs is remarkable – which prompted us to show these data and discuss what might have caused the attrition reduction. The implication of this finding is that it corroborates similar findings in a few other studies that have shown that the curriculum design seems to influence students’ dropout rates [6,7,10,23–25]. However, little is known about the role curriculum designs play in dropping out [26]. We believe the insights that we came up with in this case may fill part of this gap and could be transferred to other contexts in which dropping out is a serious problem.

After a debate among the authors – and considering the dropout percentage results, the differences between the traditional and the integrated curricula and our experiences as teachers, curriculum designers and administrative staff before, during and after the reform – we think some factors contributed to decreasing the dropout percentage in our medical school after the curriculum changed. These factors included (a) immersing students from day one in the authentic professional roles of physicians, (b) reducing biomedical sciences overload by integrating it with clinical sciences and arranging the curriculum around organ systems, (c) introducing active learning methodologies in ILAS, (d) changing the assessment system by testing the accuracy of tools (reliability and validity), (e) incorporating procedures for remediation when assessment identifies poor performance, (f) creating mentorship, and (g) the peer tutoring programs. One could assume that the changes in the structure of the curriculum boosted students’ learning and, thus, increased the chances of academic success and retention. However, in our view, the decreasing dropout percentage was not by default the direct result of the curriculum structural changes. Behind this phenomenon, hidden, lay the need to change the power relationships between teachers and students.

Specifically, it was assumed that teachers had greater knowledge and expertise than their students. This assumption may have created (in this case) an asymmetry in the power relationships between teachers and students, which we think was manifested in the vertical relationships (e.g., teachers-above, learners-below), the limited participation of students in lectures and the instrumental use of assessments to control students’ behavior. For instance, after lectures it was usual for an impromptu assessment to be made on the students if the teacher felt that they had not paid sufficient attention to what he/she said. Short, unannounced assessments with no control of validation and reliability were regularly used as methods to impose teachers’ beliefs about what a ‘well-behaved’ student was. We interpret this example as a part of the hidden curriculum in which other beliefs coexisted, such as ‘avoiding student academic failure is not a teacher’s responsibility.’ Assuming this responsibility could have been interpreted as a way to encourage students’ misbehavior in lectures, which could have generated a rupture in the verticality of relationships.

When the integrated curriculum was implemented it not only changed the arrangement of the syllabus, but also the principles of teaching and learning. Educational principles now include the premise that learners are the center of the learning process, learning activities are designed based on learning outcomes, and the role of the teacher is to guide the student to achieve those outcomes. This change of paradigm included new assumptions; for example, the teacher has the responsibility to help students who are not achieving the learning outcomes. Learners can now be rescued when they are failing academically because the power relationships with their teachers became horizontal. Assessments are no longer a tool for controlling students’ behavior but for checking if they demonstrate the competences established in the courses. Thus, students’ assessment results guide teachers to help them toward academic success. We believe this shift of educational assumptions, which the curriculum reform brought to our medical school, explains the reduction of the dropout percentage.

As a normal reaction to change, a significant level of criticism concerning the new curriculum design emerged during its implementation. Several teachers, as well as many students, demanded more control on assessment, which means increasing the difficulty level of the assessments. In some opportunities, detractors mercilessly derided the current curriculum design by spreading, for example, the idea that assessments were ‘too easy’ for students, ‘so many bad students will pass the exams and that situation will give a bad reputation to our medical school.’ Despite the criticism, there is no evidence that students from the integrated curriculum perform worse than those of the traditional curriculum in clinical skills or external standardized tests. Indeed, reactions against the current curriculum design show the political implications of the curriculum that are closely linked to the dynamics of the power relationships between teachers and students.

To sum up, based on these case findings we think that exploring the political implications of the curriculum design is key to preventing students’ dropping out. More specifically, taking into consideration the asymmetries of power relationships between teachers and students and promoting the principle that rescuing students who are failing academically is in fact a teachers’ responsibility are fundamental to retaining students. Finally, we encourage future qualitative research in order to be able to understand the role of curriculum design in dropout rates and unmask how the political dynamics embedded in the curriculum influence students’ retention.
